# Haematology and plasma biochemistry reference intervals for the critically endangered western Santa Cruz Galapagos tortoise (C*helonoidis porteri*)

**DOI:** 10.1093/conphys/coab019

**Published:** 2021-04-24

**Authors:** Ainoa Nieto-Claudín, Jamie L Palmer, Fernando Esperón, Sharon L Deem

**Affiliations:** 1 Charles Darwin Foundation, Charles Darwin Ave., Santa Cruz, 200350, Galapagos Islands, Ecuador; 2 Saint Louis Zoo Institute for Conservation Medicine, One Government Drive, Saint Louis, MO 63110, USA; 3 Complutense University of Madrid, Veterinary Faculty, Puerta de Hierro Ave, Madrid, 28040, Spain; 4 INIA-CISA, Algete-El Casar Road, Valdeolmos, 28130, Spain

**Keywords:** Baseline, giant tortoises, health, reference values, wildlife surveillance

## Abstract

Reference intervals (RIs) are an increasingly valuable tool for monitoring captive and free-living wildlife populations. Galapagos tortoises are one of the most emblematic species on Earth with 9 of the 12 extant species considered endangered due to human activities. As part of a long-term health assessment within the Galapagos Tortoise Movement Ecology Programme, we sampled a total of 210 free-living Santa Cruz Galapagos tortoises (*Chelonoidis porteri*). We collected blood from the brachial vein and performed packed cell volume (PCV), total solids (TS), morphological evaluation, white blood cell (WBC) count estimates and differentials and a VetScan biochemistry panel for each individual. We calculated 95% RIs and 90% confidence intervals (CIs) using the Reference Value Advisor tool and following international standard guidelines. Tortoises were categorized by estimated age and sex, with RI and CI reported here for 164 adult tortoises and 46 sub-adult tortoises. We found significant differences between sexes, with adult females presenting a lower PCV and higher values for both calcium and potassium. Among age groups, adult tortoises presented higher PCV, TS and albumin and lower WBC counts, aspartate aminotransferase and creatine kinase than sub-adult tortoises. We also found that tortoises presented higher numbers of lymphocytes during the dry season, but higher basophils, eosinophils, phosphorus, potassium and TS during the humid season. The heterophil:lymphocyte ratio did not differ between groups. To the authors’ knowledge, this is the first report of formal plasma biochemistry and haematology RI for free-living Galapagos tortoises. With the present study we provide an important diagnostic tool for captive-breeding programs in the Galapagos and zoological institutions globally that care for giant tortoises. The ultimate goal of this study is to contribute to the understanding of giant tortoise baseline health parameters and to inform local management decisions that help to conserve these emblematic species.

## Introduction

Reference values are typically reported as reference intervals (RIs) comprising 95% of a healthy reference population (Dybkœr and Solberg, 1987). Since their introduction for human medicine in 1969, population-based RIs have become one of the most common tools used in clinical decision-making processes for both human and veterinary medicine (Friedrichs et al., 2012; Siest et al., 2013).

Guidelines specifically addressing veterinary species have numerous benefits to the veterinary medical community. In response to this need, the American Society of Veterinary Clinical Pathology (ASVCP) formed a subcommittee to generate guidelines for de novo determination of RI in veterinary species (available on www.asvcp.org). The ultimate goal was to develop balanced and practical recommendations that are statistically and clinically valid (Friedrichs et al., 2012).

*In situ* health evaluations of wildlife species, including reptiles, have been shown to be a valuable tool for monitoring free-living populations and reintroduction programs (Deem *et al.*, 2001, 2011; Lecq *et al.*, 2014). Health evaluations often include physical examination data along with complete blood cell counts, morphological evaluation and biochemical analysis, which provide important information about the health of individuals, as well as the population being studied (Deem *et al.*, 2009; Atkins *et al.*, 2010; Boers *et al.*, 2020). Due to the logistical difficulties of describing RIs for all wildlife species, using RIs from a taxonomically related species may be helpful in some situations; however, conclusions should be drawn with extreme caution due to wide variation among species (Heatley and Russell, 2019).

The Galapagos Islands retain most of their original biodiversity, with a total of 58 reptile species described within the archipelago (Arteaga *et al*., 2019). The most iconic and emblematic species are the giant Galapagos tortoises (*Chelonoidis* spp.). Over the past few decades, the taxonomy of Galapagos tortoises has undergone a number of major shifts and revisions; however, it is currently accepted by the international union for conservation of nature (IUCN) that 14 different species once inhabited the archipelago (2 extinct, 6 critically endangered, 3 endangered and 3 vulnerable) (IUCN, 2020). The western Santa Cruz tortoise (*Chelonoidis porteri*) is native to Santa Cruz Island, the most human-populated island of the Galapagos archipelago. Whereas the estimated population for this species is considered as 3400 individuals by the IUCN (Cayot *et al*., 2017), no census has been conducted in the past decade. Based on our current studies, we estimate a population that exceeds 6000 individuals (Blake *et al*., unpublished data).

Much research has been conducted to assess the ecology, biology and genetics of giant tortoises, whereas very little is known about the health status of these species. Current threats include habitat loss and fragmentation, illegal trade, introduced and invasive species, global warming, egg loss due to introduced predators, disease, trauma and possibly antimicrobial resistance (Blake *et al.*, 2012; Ellis-Soto *et al.*, 2017; Bastille-Rousseau *et al.*, 2019; Nieto-Claudin *et al.*, 2019). Several studies have been published to describe RI for chelonian species globally; however, there was only one study on giant tortoises describing haematology and blood biochemistry parameters for 32 giant tortoises (*Chelonoidis chathamensis*) at a captive-breeding facility on San Cristóbal Island (Lewbart *et al.*, 2018), and no morphological evaluation of blood cells was documented. RIs of animals under human care are increasingly available and of great value (Species360, 2021). However, RIs calculated for free-living populations are based on animals in a more natural state with diet and other environmental factors often different from what may be provided to collection animals.

For this study, we calculated biochemical and haematology RI and described cell morphology along with morphometric measurements, body weight and body condition index from 164 free-living adults and 46 sub-adults of the Western Santa Cruz tortoise (*C. porteri*). To the authors’ knowledge, this is the first publication of formal RI of plasma biochemistry and haematology for any free-living Galapagos tortoises. The present study contributes to the understanding of giant tortoise baseline health parameters, as well as provides an important diagnostic resource for veterinarians and researchers at zoological and private collections to captive-breeding programs and local wildlife rescue networks.

## Materials and methods

### Study site

**Figure 1:**
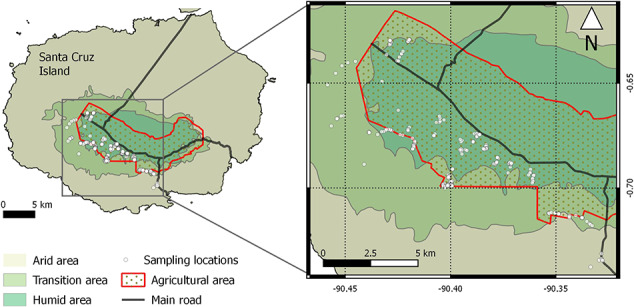
Sampling locations of 210 free-living Galapagos tortoise (*C. porteri*) on Santa Cruz Island, Galapagos.

The study site, Santa Cruz Island, is an extinct volcano that rises to a maximum elevation of 860 m in the Galapagos Archipelago. Santa Cruz is the most human-populated island of the archipelago, although almost 90% of its territory is considered national park. Tortoises were randomly sampled from different locations along their seasonal migration routes including humid and transitional habitats throughout the national park (protected areas), touristic reserves, rural (agricultural, livestock) and urban (within Puerto Ayora town limits) zones (Fig. 1). We classified those sampling areas along a gradient of anthropogenic impacts (park, tourism, urban and farming), with ‘park’ the most protected area and ‘farming’ and ‘urban’ the most impacted by human activities.

### Sampling design and collection

We collected tortoise biological samples as part of a long-term health assessment within the Galapagos Tortoise Movement Ecology Programme. From 2017 to 2020, we sampled 210 free-ranging western Santa Cruz tortoises for several health research purposes.

To facilitate animal handling, we randomly selected individuals weighing less than 200 kg. We recorded physical condition and morphometric measurements using a flexible 150-cm measuring tape. Measurements included curved carapace length (CCL), curved carapace width, curved plastron length, curved plastron width and tail length (TL). In addition, we used a hanging scale to weigh each tortoise with a precision of ±0.5 kg. We performed a physical examination and determined the sex in mature animals by tail length and plastron concavity. We classified immature animals (sub-adults) based on CCL. Despite the number of scientific publications on Galapagos tortoises, none have established a size-based age categorization for these species. For this reason, we determined age categories based on our 11 years of fieldwork experience with giant tortoises as follows: a CCL of <75 cm for immature females and <80 cm for immature males. We calculated the body condition index (BCI) as follows: BCI = m/L^2.89^ where m is mass (kg) of the tortoise and L is CCL (cm) (Blake et al., 2015).

We collected up to 5 mL of blood from the brachial vein of each tortoise with a 6-mL heparinized syringe and a 20-G 1.5-inch needle, and we made two blood films immediately at the field site for complete and differential blood cell counts (Sheldon *et al.*, 2016). We fixed blood films for 2 min by using high-quality methanol (Fixative 1, JorvetTM Diff Quick Stain Kit, Jorgensen Laboratories, USA), air dried, labelled with tortoise ID and stored in a slide box. Up to 3 mL of blood was immediately transferred to a lithium heparin tube to avoid clotting and the remaining blood was placed in a sterile cryovial. We kept heparinized blood at 4°C until analysis within the same day. We also collected a number of other biomaterials (e.g. faeces, oral, cloacal and ocular swabs, carapace scrapes, blood on Whatman® FTA card) as part of an on-going health assessment of Galapagos tortoises across islands and species. We identified tortoises by microchips previously placed by Galapagos National Park Service rangers. If no microchip was detected, we placed a new subcutaneous microchip (DATAMARS®) in the caudo-ventral area of the left hind leg.

### Haematology and plasma biochemistry

We performed packed cell volume (PCV) and total solids (TS) from heparinized blood samples within 8 hr of sample collection. PCV was determined using high-speed centrifugation of blood-filled microhematocrit tubes and manual TS via plasma analysis with a clinical refractometer (J-351, Jorgensen Laboratories, USA). We immediately separated plasma by high-speed centrifugation (1500 g, 10 min) and kept frozen at −20 C until biochemistry was performed within 2 weeks of collection.

We stained all blood films with a modified Wright Giemsa stain (JorvetTM Diff Quick Stain Kit, Jorgensen Laboratories, USA) following manufacturer’s instructions. While mammalian haematology can be performed using automated cell counters, reptilian haematologic analyses require manual methods that are technically challenging and more imprecise (Stacy *et al.*, 2011). Each method has a different set of benefits and limitations; however, estimates from blood films method is inexpensive and allows for the observation of leukocyte abnormalities such as toxic heterophils or intra-cellular inclusions (Heatley and Russel, 2019; Winter *et al.*, 2019). To reduce inter-analyses variability and inaccuracy, a well-trained technician (J.P.) performed all leukocyte quantification methods, leukocyte differentials and morphological evaluations. We determined the estimated total white blood cell (WBC) counts on the highest quality field blood film from each tortoise. WBC counts were read at 40× magnification on 10 different fields of the monolayer and averaged using the following equation: WBC (×10^3^ cells/μl) = (AVG 10 field on 40×)*1.6 (Sheldon *et al.*, 2016). We performed WBC differential counts by examining 100 WBCs on the same blood film at 100× magnification. We calculated estimated values (%) for heterophils, lymphocytes, monocytes, eosinophils and basophils. We determined heterophil:lymphocyte ratio (H:L) from the differential. Absolute values (ABS) for each white cell morphotype were calculated as follows: ABS WBC Diff (×10^9^ cells/L) = [ABS WBC (×10^9^ cells/L)] *[WBS Diff (%)] *0.01. Blood cell morphology evaluation is an essential part of the WBC assessment and can be used to identify evidence of cell toxicity and pathogen presence (mainly parasites) (Stacy *et al.*, 2011). Modified Wright-Giemsa stain, though useful and easy to use in the field, can lead to damage of cells and under-staining of some cell characteristics (Campbell, 2006). Diff Quick was still our staining method of choice due to its ease of use in remote field areas.

We performed biochemistry (Avian/Reptilian VetScan® Profile) on frozen-thawed plasma. The biochemical panel included albumin (Alb), bile acids (BA), aspartate aminotransferase (AST), calcium (Ca), creatine kinase (CK), glucose (GLU), potassium (K), sodium (Na), phosphorus (P), globulin (Glob), total protein (TP) and uric acid (UA).

We collected all samples under the Galapagos National Park annual research permits PC-36-17, PC-35-18, PC-16-19 and PC-28-20 and the International Animal Care and Use Committee from the Group of Rehabilitation of Endemic Wildlife Species (GREFA-Spain) with registration number 17/001. All samples were processed and analysed at the Charles Darwin Research Station. We performed WBC counts at the Saint Louis Zoo Institute for Conservation Medicine. CITES permits 18EC000001/PG and 19EC000001/PG and importation CITES permits 18US62698C/9 and 19US62698C/9 were used for the transportation of samples out of Ecuador.

### Statistical analyses

We used Reference Value Advisor (RefVal) v.2.1 to perform descriptive statistics (mean, median, SD, min and max) and computed 95% RI and 90% confidence intervals (CIs) for each variable. We followed ASVCP recommendations, which are based on guidelines by the International Federation for Clinical Chemistry and the Clinical and Laboratory Standards Institute (Flatland *et al*., 2013). According to the ASVCP, non-parametric methods are recommended for RI calculations when ≥120 reference samples are available and do not require assumption of a particular distribution of the data. Therefore, we used non-parametric methods to report RI and CI of adult tortoise variables when there are ≥120 reference samples. Although robust methods are preferred when there are <120 reference values, nonparametric methods can be used when the distribution of reference data is not Gaussian (Flatland *et al.*, 2013). For variables with sample sizes <120, we provide RI and CI using untransformed robust methods for Gaussian distributed data and non-parametric or Box–Cox-transformed robust methods for non-Gaussian distributed data.

We assessed symmetry and distribution of data using the Anderson–Darling test, whereby *P* ≥ 0.05 was considered to be indicative of normality and symmetry, and by visual evaluation of data histograms fitted with a Gaussian distribution curve. We evaluated the distribution of each physical measurement, haematologic and plasma biochemistry variable separately for the entire sample population, age group and sex. We identified outlier values by RefVal using Dixon and Tukey’s range tests. We closely analysed every suspected outlier and manually removed only those in which we could attribute the cause due to poor sample quality or analytic error. The ASVCP guidelines recommend partitioning the sample population into subgroups for RI and CI only if there are ≥40 or if there are clear clinical reasons. We report RI and CI separately for our total sample population of adults and sub-adult tortoises.

According to whether distribution was normal (N) or non-normal (NN), respectively, we used ANOVA (one-way analyses of variance) or Mann–Whitney *U* Test (Wilcoxon Rank Sum Test) to test for differences in all plasma biochemistry and haematology parameters between age and sex classes, sampling season and location. Additionally, we took the same approach, using ANOVA or Mann–Whitney *U* to test for differences in plasma biochemistry and haematology parameters between grades of hemolysis (considering Grade 1 as Hemo ≤1 and Grade 2 as Hemo >1 based on VetScan reports). We performed the analyses in R 4.0.2 and IBM® SPSS Statistics 25.

## Results

Based on our morphological classification, we sampled 46 (30.5%) sub-adults and 164 (78%) adult tortoises [111 (67.7%) females and 53 (32.3%) males]. All individuals were determined to be in good health based on physical evaluations and BCI score, considering a BCI of >0.1 as acceptable (Blake *et al.*, 2015) and having an average BCI of 0.14 for adults and sub-adults. There was a significant difference in body mass and length between sexes with males having an average CCL of 109.5 ± 17.9 cm and weight of 102.1 ± 33.8 kg, whereas females had an average CCL of 91.2 ± 9.2 and weighed 69.5 ± 21.8 kg (*P* < 0.001). Tortoise tail length also varied by sex with males having an average TL of 9.2 ± 3.5 cm and females an average TL of 4.9 ± 1.1 cm (*P* < 0.001). Sub-adult individuals were significantly smaller than adults with an average CCL of 67.8 ± 6.5 cm, TL of 4.1 ± 0.8 cm and weighed 29.7 ± 7.2 kg (*P* < 0.001).

We performed manual PCV and TS for all 210 blood samples, although only 159 (75.7%) individuals were examined for WBC estimate and differentials due to difficult field and weather conditions that caused blood smears to have poor cell quality.

In Table 1, we report nonparametric RI and CI of haematology variables in adult tortoises. In Table 2, we report RI and CI for sub-adult tortoises and the method used for calculations.

**Table 1:** Nonparametric RIs and CIs of haematology parameters for western Santa Cruz adult tortoises (*C. porteri*)

Analyte	SI units	*N*	Mean	Median	±SD	Min	Max	LRI	URI	90% LCI	90% UCI	Distrib.
PCV	%	156	21.1	22.0	3.0	14	28	14	26.1	14.0–16.0	26.0–28.0	NN
WBC	x10^9^/L	127	19.4	17.1	9.2	5.6	42.2	6.6	40.3	5.6–7.0	37.6–42.2	NN
Heterophils	%	127	11.5	9.0	7.1	1	39	2.2	29.8	1.0–3.0	23.0–39.0	NN
ABS het	x10^9^/L	127	2.0	1.7	1.3	0.2	6.1	0.3	5.3	0.2–0.5	4.6–6.1	NN
Lymphocytes	%	127	79.3	82.0	10.5	47	96	55.0	94.8	47.0–58.0	93.0–96.0	NN
ABS lymph	x10^9^/L	127	15.8	13.8	8.6	3	38	4.5	36.3	3.0–5.1	32.3–38.0	NN
Monocytes	%	127	1.6	1.0	1.5	0	6	0.0	5.0	0.0–0.0	5.0–6.0	NN
ABS mono	x10^9^/L	127	0.3	0.2	0.3	0	1.6	0.0	1.2	0.0–0.0	0.9–1.6	NN
Eosinophils	%	127	3.3	2.0	4.3	0	20	0.0	17.0	0.0–0.0	12.0–20.0	NN
ABS eos	x10^9^/L	127	0.5	0.3	0.6	0	2.8	0.0	2.2	0.0–0.0	1.7–2.8	NN
Basophils	%	127	4.3	4.0	2.7	0	11	0.0	10.0	0.0–0.0	9.0–11.0	NN
ABS baso	x10^9^/L	127	0.7	0.6	0.5	0	2.8	0.0	2.1	0.0–0.0	1.7–2.8	NN
H:L ratio		127	0.2	0.1	0.1	0.01	0.67	0.0	0.5	0.0–0.0	0.4–0.7	NN

**Table 2:** RIs and CIs of haematology parameters for western Santa Cruz sub-adult tortoises (*C. porteri*) and the method used for calculations

Analyte	SI units	*N*	Mean	Median	±SD	Min	Max	LRI	URI	90% LCI	90% UCI	Distrib.
PCV	%	46	18.6	19.0	4,0	10	26	10.0	26	10.0–12.4	24.1–26.0	N
WBC^a^	x10^9^/L	32	25.7	26.9	10.6	4.2	45.6	4.6	49.1	1.0–9-6	43.6–54.0	N
Heterophils^a^	%	30	9.2	9.0	5.0	1	19	0.6	21.4	0.0–2.4	18.2–24.5	N
ABS het^a^	x10^9^/L	30	2.3	2.3	1.3	0.2	5.1	0.0	5.2	0.0–0.2	4.4–5.9	N
Lymphocytes^b^	%	30	84.5	85.0	6.7	68	96	68.7	96.7	63.0–73.7	94.0–99.4	N
ABS lymph^b^	x10^9^/L	30	22.5	24.9	9.2	6.0	40.1	5.4	43.5	2.3–9.2	37.6–49.8	N
Monocytes^c^	%	30	1.3	1.0	1.0	0	3	0.0	3.4	0.0–0.0	2.9–3.8	NN
ABS mono^d^	x10^9^/L	30	0.3	0.3	0.3	0	1.2	0.0	1.0	0.0–0.0	0.8–1.2	NN
Eosinophils^c^	%	30	1.8	1.0	2.3	0	9	0.0	6.6	0.0–0.0	4.6–8.4	NN
ABS eos^c^	x10^9^/L	30	0.4	0.2	0.6	0	2.0	0.0	1.6	0.0–0.0	1.0–2.0	NN
Basophils^c^	%	30	3.2	3.0	2.1	0	9	0.0	7.5	0.0–0.0	6.1–8.8	NN
ABS baso^d^	x10^9^/L	30	0.8	0.6	0.7	0	3.4	0.0	2.2	0.0–0.0	1.5–2.8	NN
H:L ratio^a^		30	0.1	0.1	0.1	0.01	0.26	0.0	0.3	0.0–0.0	0.2–0.3	N

The sample size was too small (*n* < 40) to calculate a nonparametric RI for some parameters. Alternative statistical methods were used after checking the symmetry of the distribution.

a ^a^Robust method with a Box-Cox transformation.

b ^b^Standard method with a Box-Cox transformation.

c ^c^Untransformed standard method.

d ^d^Untransformed robust method.

We found significant statistical differences among male and female adult tortoises for PCV (*P* < 0.001) with males presenting higher haematocrit than females (mean PCV: 20.7% for females and 22% for males). Likewise, we found significant statistical differences among age groups for manual PCV (*P* < 0.001) and TS (*P* < 0.001), with adults having higher values for both parameters. We also found differences among adults and sub-adult tortoises for WBC estimates (*P* = 0.002), with adults having lower WBC when compared to sub-adults, and for lymphocytes (*P* = 0.026) and basophils (*P* = 0.021), with adults having slightly fewer lymphocytes and more basophils than sub-adults. The H:L was lower in subadult tortoises (0–0.3) when compared to adults (0–0.5) but this difference was not significant. H:L did not differ between the sexes.

**Figure 2:**
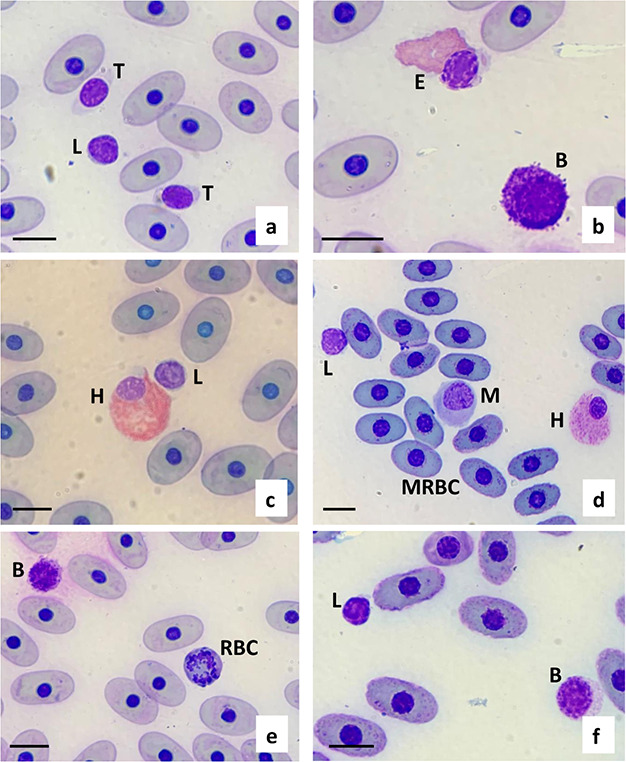
Modified Wright-Giemsa stained peripheral blood from a Galapagos tortoise (*C. porteri*) showing L, lymphocyte; T, thrombocyte; H, heterophil; B, basophil; M, monocyte (most likely azurophil); E, eosinophil; MRBC, mature red blood cell with basophilic inclusions in the cytoplasm; RBC, immature mitotic red blood cell (size bar, 10 μm).

There were significant differences for some haematological parameters based on sampling season. Tortoises of all ages presented a higher number of lymphocytes during the dry season (*P* = 0.009) and a higher number of basophils during the humid season (*P* = 0.002). Adult tortoises also showed a slightly higher number of eosinophils during the humid season (*P* = 0.017). The main morphological characteristics of the blood cells are provided in Fig. 2. Drying artefacts were present in approximately half of the blood slides, which we attributed to poor field conditions, including rain and humidity, which can both affect cell quality and lead to slide artefacts. Mature erythrocytes were oval with dense chromatin in the nucleus and basophilic inclusions in some cases (Fig. 2d). Vacuoles in the cytoplasm of the erythrocytes were observed, although infrequently. Immature red blood cells (RBCs) were also infrequently observed, smaller and rounder, with a basophilic cytoplasm, less dense chromatin in the nucleus and some mitotic figures (Fig. 2e). Lymphocytes were the most predominant leukocyte (79.3 ± 10.5). The nucleus was dark with dense, clumped chromatin and cytoplasm ranged from light to dark blue in colour. In most cases, the lymphocytes were easy to distinguish from thrombocytes as thrombocyte cytoplasm was clear to light blue and the nucleus was typically elongated, and chromatin looked smooth (Fig. 2a). Lymphocyte-like cells where the cytoplasm could not be differentiated from thrombocytes were not included in the total WBC counts or differentials. Heterophils were the second most predominant leukocyte (11.5 ± 7.1) The nucleus was round or lobed, eccentric, with a slightly clear cytoplasm and rod shaped, pink and glass-like refractive granules (Fig. 2c and d). In some tortoises, the granules were fused and therefore difficult to observe. In all tortoises, most heterophils had light grey, circular intracytoplasmic inclusions. This characteristic helped with differentiation between heterophils and eosinophils. Nuclei in eosinophils were typically round, eccentric and dark with grey to clear cytoplasm. Granules were round, less densely packed and stained darker than heterophil granules, ranging from dark pink to purple grey (Fig. 2b). Monocytes were identified by their cytoplasm to nucleus ratios, larger, often kidney-shaped nucleus and presence of cytoplasmic vacuoles. Azurophils were counted as monocytes for the differential as is recommended for all reptiles except snake species (Stacy *et al.*, 2011). Azurophils were morphologically difficult to distinguish from monocytes (Fig. 2d). Basophils were small, with densely packed dark purple granules throughout the cytoplasm often covering the nucleus. The nuclei were dark purple and difficult to distinguish from the granules in the cytoplasm. Some basophils showed a degranulated image (Fig. 2b, c and f). No structures compatible with hemoparasites were observed.

We ran a total of 210 Vetscan avian/reptilian panels, with 3 (1.4%) panels cancelled due to internal quality issues detected by the Vetscan VS2 analyser. The system also reported 52 (24.8%) instances in which one or more analyte was outside of the linear range of the analyser, but the remaining analytes were correctly analysed and reported. Typically, this finding was noted in the Alb, Glob and/or CK analytes. For Ca and K, several samples were reported beyond the detection range of the analyser. A total of 12 samples reported Ca > 16 mg/dl, whereas 7 samples reported Ca > 20 mg/dl. All 19 samples corresponded to adult females and only 3 showed hemolysis of >1. A total of 54 samples reported K > 8.5 mmol/L (41 adults and 13 subadults) with 64.8% (35/54) of these results corresponding to hemolytic samples (hemolysis > 1). For these two instances (Ca and K) and in order to calculate the RI, we were very conservative and gave the closest numeric value (16.1 mg/dl for Ca > 16, 20.1 mg/dl for Ca > 20 and 8.6 mmol/L for K > 8.5). Bile acids were not calculated by the VetScan analyser and were reported as <35 μmol/L in all 210 samples so we were not able to calculate RI for this analyte.

We found statistically significant differences between grades of hemolysis for several plasma chemistry variables. However, RI calculations using all samples vs. only hemolysis grade 1 samples computed the same intervals for all analytes except K. For this reason, we report RI and CI using the total number of samples for all analytes but K. For K we report RI and CI only for samples with hemolysis of ≤1. In Tables 3 and 4, we report nonparametric RI and CI of plasma biochemistry variables in adult and sub-adult tortoises, respectively.

**Table 3:** Nonparametric RIs and CIs of plasma biochemistry parameters for western Santa Cruz adult tortoises (*C. porteri*)

Analyte	SI units	*N*	Mean	Median	±SD	Min	Max	LRI	URI	90% LCI	90% UCI	Distrib.
TS	mg/dL	161	6.5	6.2	1.2	4	11	4.6	9.8	4.0–4.8	8.6–11.0	NN
AST	U/L	153	46.9	44.0	15.2	22	112	24.9	84.2	22.0–27.0	78.0–112.0	NN
CK	U/L	143	2024.8	1558	1432.1	99	6124	260.0	5296.0	99.0–409.0	4953.0–6124.0	NN
UA	mg/dL	159	1.8	1.8	0.6	0.5	3.4	0.8	3.2	0.5–0.9	2.9–3.4	N
GLU	mg/dL	157	70.9	68.0	23.7	15	132	26.7	123.2	15.0–35.0	117.0–132.0	N
CA	mg/dL	145	12.0	11.4	2.6	6.8	20.1	7.8	20.1	6.8–9.0	16.1–20.1	NN
FOS	mg/dL	155	4.1	4.1	0.6	2.7	5.8	3.2	5.4	2.7–3.3	5.2–5.8	NN
TP	g/dL	160	5.9	5.8	0.9	3	8.4	3.7	7.7	3.0–4.5	7.4–8.4	N
ALB	g/dL	147	1.6	1.6	0.4	1	2.6	1.1	2.5	1.0–1.1	2.3–2.6	NN
GLOB	g/dL	145	4.3	4.2	0.8	2.4	6.8	2.9	5.9	2.4–3.1	5.5–6.8	N
NA	mmol/L	151	126.6	126.0	5.2	114	144	116.8	138.2	114.0–119.0	135.0–144.0	NN
K	mmol/L	159	7.6	7.7	1	3.3	8.6	5.3	8.6	3.3–5.9	8.6–8.6	NN
K^a^	mmol/L	81	7.4	7.5	1.1	3.3	8.6	4.1	8.6	3.3–5.4	8.6–8.6	NN

a ^a^Potassium RI calculated only for samples with hemolysis ≤1.

**Table 4:** Nonparametric RIs and CIs of plasma biochemistry parameters for western Santa Cruz sub-adult tortoises (*C. porteri*)

Analyte	SI units	*N*	Mean	Median	±SD	Min	Max	LRI	URI	90% LCI	90% UCI	Distrib.
TS	mg/dL	46	5.4	5.5	1	3.4	7.2	3.4	7.2	3.4–4.2	7.0–7.2	N
AST	U/L	44	54.6	51	16.8	31	96	31.0	95.5	31.0–32.4	82.0–96.0	N
CK	U/L	41	2534.8	2286	1321.4	556	5227	56.1	5225.0	556.0–899.2	4632.0–5227.0	N
UA	mg/dL	46	1.9	1.8	0.7	0.7	4.2	0.7	4.0	0.7–0.8	2.7–4.2	N
GLU	mg/dL	45	76.2	79.0	26.3	32	131	32.5	130.9	32.0–36.9	121.0–131.0	N
CA	mg/dL	45	11.6	11.6	1.3	6.8	14	7.2	13.9	6.8–10.0	13.4–14.0	N
FOS	mg/dL	46	4.1	4.1	0.6	2.8	5.4	2.8	5.4	2.8–3.5	4.8–5.4	N
TP	g/dL	46	5.1	5.1	0.9	2.5	7.1	2.7	7.0	2.5–3.8	6.1–7.1	N
ALB^a^	g/dL	26	1.3	1.3	0.3	0.7	2	0.7	1.9	0.6–0.9	1.7–2.1	N
GLOB^b^	g/dL	23	4.1	4.0	0.8	2.8	6	2.6	5.9	2.3–3.0	5.4–6.5	N
NA	mmol/L	45	127.4	128.0	5.0	117	138	117.5	137.9	117.0–120.3	136.4–138.0	N
K	mmol/L	46	7.8	7.9	0.9	4	8.9	4.4	8.8	4.0–6.7	8.6–8.9	NN

The sample size was too small (*n* < 40) to calculate a nonparametric RI for some parameters. Alternative statistical methods were used after checking the symmetry of the distribution.

a ^a^Standard method with a Box-Cox transformation.

b ^b^Robust method with a Box-Cox transformation.

We found significant statistical differences among male and female adult tortoises for Ca (*P* = 0.009) and P (*P* < 0.001) with females presenting higher values for both parameters (mean: females, Ca = 12.9 and *P* = 4.2 mg/dl; males, Ca = 11 and *P* = 3.9 mg/dl). We found differences among age groups, with adults showing higher TP (*P* < 0.001) and Alb (*P* < 0.001), and lower AST (*P* = 0.004) and CK (*P* = 0.012) than sub-adults. However, some of these differences might be due to the sample size variability (only 46 sub-adults when comparing to 164 adults).

We found significant differences for some biochemistry parameters based on sampling season. Tortoises of all ages presented higher values for P (*P* = 0.003), K (*P* = 0.035) and TS (*P* = 0.026) during the humid season.

Tortoises sampled within the national park area showed a higher AST during the humid season, when compared to the dry season (mean: 66.6 and 48.3 U/L, respectively) (*P* = 0.019). We explored differences among sampling areas and found that tortoises sampled within the national park showed higher values of AST than those sampled within farming areas during the same humid season (*P* = 0.003). By contrast, tortoises within the farming areas presented a higher Alb during the humid season when compared to the dry season (*P* < 0.001) with an average of 1.9 g/dL during the humid season and 1.5 g/dL during the dry season.

## Discussion

Despite the ecological and economic importance of giant tortoises for the Galapagos archipelago, very little is known about their health status. With this study we contribute to the understanding of giant tortoise baseline health parameters with formal RIs for the critically endangered western Santa Cruz giant tortoise (*C. porteri*).

Lewbart *et al*. (2018) reported biochemistry and haematology parameters of 32 San Cristóbal Galapagos tortoises (*C. chathamensis*), housed at a captive-breeding facility and using an iSTAT portable clinical analyser. When compared to their results, we obtained higher values for both adult and sub-adult tortoises for mean K, Glu and TS (K, 7.6 vs. 4.4 mmol/L; Glu, 70.9 vs. 45.5 mg/dL; and TS, 6.5 vs. 5.2 mg/dL, respectively, in adult tortoises). Despite the fact that their study used a different analyser and tortoise species and a much smaller sample size, results from it fit within the RI that we have calculated for Santa Cruz tortoise species.

Estimations of PCV and TS were reported by several authors in different species of giant tortoises (Knafo *et al*., 2011; Rivera *et al*., 2011; Blake *et al*., 2015; Lewbart *et al*., 2018), but sample sizes were smaller than 40 individuals in all of these studies. PCV (%) values were very similar between previous studies and the present study, whereas TS (mg/dL) differed in some of the reports with lower values in Rivera *et al*. (2011) and Lewbart *et al*. (2018). However, all results fit within the RI that we have calculated for both parameters.

WBC estimates and differentials were reported by Sheldon *et al*. (2016) in a study to compare leucocyte quantification methods. Results from their study, using the same methodology, concluded lower values for WBC estimates, absolute heterophils and lymphocytes, but report similar values for absolute monocytes and basophils and slightly higher results for absolute eosinophils. However, all Sheldon *et al.* (2016) results fit within the RI that we report here for all parameters. By contrast, Lewbart *et al*. (2018) reported WBC estimates and differentials percentages for a different species of giant tortoise (*C. chathamensis*) maintained at a captive breeding center. They used a different methodology, performing WBC counts at 10× instead of 40× magnification and multiplied by 2000 so we did not compare this with our results. Interestingly, they reported a very high percentage of heterophils (46%) and monocytes (17%), whereas we found giant tortoises to be lymphocyte dominant (79.3%) with a low percentage of monocytes (1.6%), as in agreement with Sheldon *et al*. (2016). The H:L also supports that haematology of giant tortoises is lymphocyte predominant (H:L ≤ 0.5). Differences found among studies are most likely due to species-specific reasons and/or captive vs. free-living variations.

To the author’s knowledge, this is the first complete description of erythrocyte and leukocyte morphology for free-living giant Galapagos tortoises. Inclusions, vacuoles and immature figures can be observed in reptilian RBC due to a variety of processes including sample collection method, field conditions, stress, poor slide preparation or staining and/or disease (Heatley and Russel, 2019; Rodríguez *et al*., 2018). Mitotic figures in circulating reptile RBC have been described as normal findings (Sykes and Klaphake, 2008), whereas erythrocytes in chelonians may contain basophilic cytoplasmic inclusions, thought to be degenerating organelles or haemoglobin precipitates, with no pathologic significance (Stacy *et al.*, 2011; Zhang *et al.*, 2011; Hernández *et al.*, 2016). Cytoplasmic vacuoles, mitotic and immature figures were infrequently observed in the current study, and the authors associate these findings with the poor quality of the slides and the abundance of artefacts due to the high humidity and rain during field slide preparation. Heterophil inclusions have been observed in sick captive giant tortoises with no difference upon recovery (personal communication from Dr Nicole Stacy, University of Florida). Based on our experience evaluating giant tortoise slides from tortoises deemed healthy by physical examination, and from a number of C*helonoidis* spp. and considering that light inclusions were found in most heterophils, we conclude no pathologic significance of heterophil inclusions in the hemogram.

Variability in haematology and plasma biochemistry values is known to exist both within and between reptilian species. This variation has been attributed to environmental conditions such as climate, season and toxins, as well as nutrition, age, sex, population dynamics, method of collection, sample handling and biochemical assay method (Deem *et al.*, 2009; Scope *et al.*, 2013; Boers *et al.*, 2020). This is also true for our findings on lymphocytes, eosinophils, basophils, P, K and TS, in which we report different values according to the season; as well as differences found in Alb and AST among sampling areas. Seasonal changes in plasma enzymes, electrolytes, metabolites and proteins have been described for a wide variety of reptile species (Heatley and Russel, 2019), whereas sampling location might also be correlated with different migratory patterns and different foraging strategies as demonstrated to influence tortoise fitness (Blake *et al.*, 2015). Seasonal plasma increases of total calcium and phosphorous in free-living and captive females during vitellogenesis occurs in all major reptile taxa (Deem *et al.*, 2009; Scope *et al.*, 2013, Heatley and Russel, 2019), which explains the higher results obtained in adult females. Adult reptiles tend to have greater concentrations of albumin and therefore total protein than sub-adults and juveniles (Heatley and Russel, 2019), which also supports our results of higher TP, Alb and TS in adult giant tortoises. Additionally, it is important to highlight that Galapagos tortoises do not go through hibernation as many other turtle species, and therefore we can find actively reproductive individuals year-around in Santa Cruz, with a peak mating season between November and March (humid season) and the peak nesting season between July and September. Clinical interpretation of enzyme activity remains minimally studied in reptiles. CK and AST are commonly evaluated in reptile biochemistry profiles. Increased AST may be derived in large quantities from muscle, liver, or other tissues (Heatley and Russel, 2019) or may be attributed to muscle and/or liver injury; although they may also be associated with a high level of exercise and musculoskeletal wear, as may occur in migratory tortoises.

The precision of the point-of-care Vetscan VS2 analyser has been described as accurate for some turtle species and parameters (Atkins *et al.*, 2010)*.* Based on our findings, some analytes in giant tortoises should be evaluated and/or confirmed with additional testing methods (BA, Ca, K, CK, Alb, Glob), as was also described in sea turtles and other reptile species (Gibbons *et al*., 2019). The VetScan system reported instances mainly for Alb and Glob and parameters were also affected by the quality of the sample, so we recommend additional methods when testing captive or sick individuals for these parameters. The Avian/Reptilian VetScan profile cannot be used to calculate BA in giant tortoises as the values appear to be below the lower detection limit of the analyser. Moreover, the upper detection limits of the Avian/Reptilian profile for Ca and K are below the normal values found within healthy giant tortoises, mainly reproductive females. Lastly, using the VetScan analyser, there was a poor level of precision for CK. Laboratory-based technology, when available, may be better to evaluate biochemical parameters in these species due to these limitations.

In conclusion, this study contributes to the baseline database of health parameters for Galapagos tortoises. These data may be helpful for the Galapagos National Park Service personnel in efforts to preserve these species, free-living in the National Park and at captive-breeding centers, as well as for monitoring the health and welfare of giant tortoises within private and zoological institutions around the world. Future research should focus on establishing RIs for all Galapagos tortoise species to facilitate comparisons and a deeper understanding of tortoise eco-physiology and pathology.

## Funding

This work was supported by the Saint Louis Zoo’s Field Research for Conservation (FRC) program (FRC#2018.03), Saint Louis Zoo WildCare Institute Center for Chelonian Conservation, Charles Darwin Foundation, Houston Zoo, Galapagos Conservation Trust, Ecoventura, Linda Esler and Miss Karen Lo.

## Conflict of Interest Statement

Ainoa Nieto Claudín, Jamie Palmer, Fernando Esperón, and Sharon L. Deem declare there is no conflict of interest.

We followed all institutional and national guidelines for the care and use of animals. We collected samples under the Galapagos National Park annual research permits PC-36-17, PC-35-18, PC-16-19 and PC-28-20 and the International Animal Care and Use Committee from GREFA, Spain, with registration number 17/001. All samples were processed and analyzed at the Charles Darwin Research Station. We performed WBC at the Saint Louis Zoo Institute for Conservation Medicine. We transported samples under exportation CITES permits 18EC000001/PG and 19EC000001/PG and importation CITES permits 18US62698C/9 and 19US62698C/9.
